# Tumor Microenvironment and Immunotherapy Response in Head and Neck Cancer

**DOI:** 10.3390/cancers12113377

**Published:** 2020-11-15

**Authors:** Panagiota Economopoulou, Ioannis Kotsantis, Amanda Psyrri

**Affiliations:** Section of Medical Oncology, Department of Internal Medicine, National and Kapodistrian University of Athens, Attikon University Hospital, 12462 Athens, Greece; panagiota_oiko@hotmail.com (P.E.); ikotsantis@gmail.com (I.K.)

**Keywords:** tumor microenvironment, immunotherapy, head and neck cancer, tumor-associated macrophages, cancer-associated fibroblasts

## Abstract

**Simple Summary:**

Immunotherapy has revolutionized cancer treatment and has been integrated in the treatment algorithm of metastatic head and neck cancer. Despite robust clinical efficacy shown in clinical trials, only a minority of patients derive benefit from immunotherapy. Indeed, an important parameter that affects the effectiveness of immunotherapeutic drugs is the tumor microenvironment (TME), whose cellular elements participate in tumor evolution and metastasis. Through interaction with TME cells, tumor cells have the capacity to generate an immunosuppressive TME that may substantially influence the response to immunotherapy. In this review, we aim to illustrate the complex interplay between TME cells and describe their potential role as therapeutic targets with the goal to overcome treatment resistance.

**Abstract:**

The tumor microenvironment (TME) encompasses cellular and non-cellular components which play an important role in tumor evolution, invasion, and metastasis. A complicated interplay between tumor cells and adjacent TME cells, such as stromal cells, immune cells, inflammatory cells, and cytokines, leads to severe immunosuppression and the proliferation of cancer cells in several solid tumors. An immunosuppressive TME has a significant impact on treatment resistance and may guide response to immunotherapy. In head and neck cancer (HNC), immunotherapeutic drugs have been incorporated in everyday clinical practice. However, despite an exceptional rate of durable responses, only a low percentage of patients respond. In this review, we will focus on the complex interactions occurring in this dynamic system, the TME, which orchestrate key events that lead to tumor progression, immune escape, and resistance. Furthermore, we will summarize current clinical trials that depict the TME as a potential therapeutic target for improved patient selection.

## 1. Introduction

Head and neck squamous cell carcinoma (HNSCC) is a heterogeneous disease with a typically dismal prognosis in the recurrent/metastatic (R/M) setting [1]. T-cell based immunotherapies, such as immune checkpoint inhibitors (ICIs), have been shown to improve overall survival (OS) in R/M HNSCC. ICIs interfere with the host’s immune response by re-activating cytotoxic T cells and rely on their capacity to eradicate tumor cells [2]. Given that approximately 20% of patients with R/M HNSCC respond to the approved immunotherapeutic drugs pembrolizumab and nivolumab when administered as monotherapy [3,4], it is essential to decode the signs of response to immunotherapy in order to reduce treatment costs and spare patients from severe autoimmune side effects. Indeed, it is known that the immunotherapy response can be modified by the tumor’s genetic profile and the host’s immune behavior.

Compelling evidence from preclinical and clinical studies suggests that cancer cells have the ability to create an immunosuppressive tumor microenvironment (TME) and that this tumor-mediated immunosuppression might decisively affect immune response and treatment effectiveness [5]. HNSCC is characterized by the compromised function of natural killer (NK) cells, lower lymphocyte counts in patients with active disease as compared to healthy individuals, disruption of the antigen presenting machinery, and impaired activity of tumor-infiltrating lymphocytes (TILs) [6,7,8]. Therefore, immunotherapeutic strategies face the task of overcoming barriers of immune suppression in patients with HNSCC. A greater understanding of the intricate interaction between tumor cells and cells in the TME will shed light on factors contributing to immunotherapy resistance and further improve the efficacy of immunomodulatory approaches for HNSCC.

In this review, we will summarize the factors that shape the immune response and focus on immune suppressive signals of the TME as determinants of immunotherapy efficacy.

## 2. Development of Anti-Tumor Immune Response

In 1909, Elrich suggested the theory of cancer immunosurveillance, which was based on the notion that the host’s immune system has the capacity to recognize, control and eliminate growing tumors [9]. This theory was later developed by Burnet and Thomas, who proposed the idea of diversity in tumor antigens as compared to normal cells [10]. Although this hypothesis was rejected for a number of years, several key findings in murine experimental models led scientists to invigorate the idea, such as the discovery of antitumor effects of interferon-gamma (IFN-γ), lymphocytes, and NK cells [11,12]. Moreover, it was observed that immunocompromised mice, e.g., depleted of IFN-γ responsiveness, were more susceptible to the formation of malignant tumors [11,13]. Therefore, tumors that evolve in the context of immunodeficiency have the tendency to be more immunogenic [13]. Subsequently, several observations in humans, such as an elevated risk of cancers in transplant patients who are subjected to long-term immunosuppression [14], resulted in the scientific confirmation of the immunosurveillance theory.

A dynamic anti-tumor response is generated during the process of immune surveillance. Indeed, T cells have the capacity to identify cancer cells following a process of tumor antigen presentation. Tumor neoantigens (TA) are products of mutant genes and represent a subclass of tumor antigens that are purportedly solely produced by the tumor cells [15]. Thus, tumors exhibit individually distinct antigen determinants (neoantigens) that can prompt a tumor-targeted immune response. HNSCC is characterized by a high frequency of p53 tumor suppressor loss, which promotes the formation of TAs and results in genomic instability favoring immunogenicity [16]. TAs are released by cancer cells and subsequently engaged by antigen-presenting cells (APCs), e.g., dendritic cells (DCs). The captured TAs are presented through a major histocompatibility complex (MHC) class I molecule to the T cell receptor (TCR) propelling the activation and trafficking of Cytotoxic T Cells (CTLs) to the tumor [17]. Finally, CTLs infiltrate tumor sites, identify and eliminate cancer cells via exocytosis of granules containing perforin and enzymes that result in tumor cell death.

The activation of T cells is regulated by immune checkpoints [18]. Co-stimulatory molecules, such as B7-1/B7-2 ligands and cluster differentiation 28 (CD28) receptor, that are expressed on the surface of APCs and T-cells, respectively, enhance T-cell activation [19]. On the contrary, inhibitory immune checkpoints, such as programmed death-1 (PD-1) and cytotoxic T-lymphocyte associated protein-4 (CTLA-4), which are co-expressed on T cells, normally block T-cell activation in order to preclude maximal immune reactions. More specifically, CTLA-4 binds to B7-1 and B7-2 ligands with greater affinity than CD28 and sends an inhibitory signal to T cells [20]. PD-1 binds to its ligands programmed cell death ligand-1 (PD-L1) and programmed cell death ligand-2 (PD-L2) and suppresses T cell activation, promoting T cell exhaustion [21].

However, certain tumor cells evolve and have the capacity to disrupt, repress or evade the immune system by interfering with the normal function of immune cells [13]. Mechanisms of immune escape in HNSCC include the formation of novel mutated antigens, disruption of the antigen-presenting machinery, overexpression of PD-L1 inhibitory molecule in CTLs, secretion of inflammatory cytokines and immunosuppressive factors (transforming growth factor-beta (TGF-β), IL-10, indoleamine 2,3-dioxygenase (IDO)) and recruitment of key immunosuppressive cell types in the TME, such as T regulatory cells (Tregs) and myeloid derived suppressor cells (MDSCs) [6,22].

## 3. The Role of TME as a Dynamic Ecosystem in HNSCC

The cellular components of the TME develop jointly with the tumor. For instance, non-malignant cells, such as macrophages and fibroblasts, alter their phenotype and transmit signals that contribute to tumor-specific properties [23]. Nevertheless, a significant determinant of response to immunotherapy is the pre-existing immunity of the TME. Gene profiling studies in melanoma and other solid tumor tissues have identified two distinct immune profiles, inflamed or non-inflamed, based on the expression of genes suggestive of immune cell infiltration [24,25,26]. Thus, inflamed tumors are defined by the presence of CTLs and other immune cells, such as myeloid cells and T-regs, IFN-γ expression, and the secretion of effector cytokines [26,27]. Several inhibitory molecules, such as PD-L1 and IDO, are upregulated in tumors with an inflamed microenvironment, mainly driven by the increased production of IFN-γ, which is caused by the increased recognition of TAs by CTLs [28,29]. In addition, CTLs often display a dysfunctional phenotype [25,30]. The immune-inflamed TME is indicative of a prior anti-tumor response that was abruptly ceased possibly due to the immunosuppressive milieu that has emerged in the tumor itself; thus, it usually represents a breeding ground for a clinical response to immune checkpoint blockade [31,32]. Solid tumors, such as lung cancer, melanoma, renal cell carcinoma, and HNSCC, are often presented with an inflamed phenotype.

On the contrary, a non-inflamed TME (excluded or immune-desert) is characterized by the absence of infiltrating CTLs, IFN-γ signature and inhibitory pathways [30]. Immunosuppressive cytokines and cellular components, such as Tregs and tumor-associated macrophages (TAMs), usually dominate [26]. More specifically, the immune-desert phenotype is utterly depleted of infiltrating T cells, precluding the existence of a prior anti-tumor response. Thus, it is proposed that immune-desert tumors, such as prostate cancer, only anecdotally respond to immune checkpoint inhibitors (ICIs) [31]. In the non-inflamed immune-excluded TME, although the presence of immune cells is documented, these remain in the tumor stroma instead of infiltrating the parenchyma. In this context, a pre-existing anti-tumor response might have been produced but blocked as inefficacious due to inability of immune cells to be transferred through the stroma [33]. Immune excluded tumors, such as colorectal and pancreatic cancer, which are characterized by abundant stroma tissue, rarely respond to immunotherapy [31].

HNSCC is presented as an inflamed tumor, characterized by the abundance of immune cells. HNSCC tumor cells struggle to generate a potent immunosuppressive TME that will contribute to cancer progression. Recruitment of cellular components such as Tregs and MDSCs is of paramount importance for immune escape.

### 3.1. Tumor-Associated Macrophages (TAMs)

Macrophages are monocytes with phagocytic properties found in tissues and based on their degree of differentiation and functional capacity are subclassified into M1 and M2 varieties, which represent the two extreme edges of a broad phenotypic range [34]. These two groups differ substantially in their functional role, which is mainly defined by their origin, location and surrounding environmental factors [35]. Thus, M1 macrophages possess proinflammatory properties and boost the antitumor immune response through the production of inflammatory cytokines, such as IL-12, IL-23, IFN-γ and reactive oxygen species [36]. The function of M2 macrophages contrasts with the anti-tumor effect of M1. Indeed, M2 macrophages, which are characterized as tumor-promoting, secrete immunosuppressive cytokines, such as IL-10 and TGF-β, propel angiogenesis, thereby favoring an immunosuppressive milieu [37,38]. M2 macrophages display increased expression of CD163, a receptor for haptoglobin-hemoglobin that has a major role in removing haptoglobin-hemoglobin adducts and promoting innate immune response [39]. CD163 has been identified as an M2 specific marker. An additional marker, CD68, is also expressed in M2 but is not highly specific.

The terminology ‘tumor- associated macrophages’ (TAMs) has been introduced to describe a population of mature macrophages recruited in the TME that usually have a M2 phenotype [40]. Recruitment of macrophages in the TME is achieved either through the bone marrow or via differentiation of MDSCs in the TME and is regulated by several hematopoietic growth factors, such as colony stimulating factor 1 (CSF1) and monocyte chemotactic protein 1 (MCP-1) and chemokines. Hypoxic conditions favor the colonization of TAMs to the TME [38,41]. Once in the TME, they have a key role in constructing a profoundly immunosuppressive milieu through a variety of mechanisms, predominantly the metabolic repression of T cells, PD-L1 expression, and other molecules, suppression of NK cells, and immunosuppressive cytokines, such as IL-6, IL-10, and TGFβ [42].

Abundance of TAMs in the TME has been shown to be an unfavorable prognostic factor in a variety of cancers, such as breast, gastric and ovarian cancer [43,44,45]. In a retrospective study that included surgical biopsies from patients with oral cavity squamous cell carcinoma (OSCC), it was found that the majority of TAMs, which were assessed by immunohistochemistry, were positive for anti-inflammatory cytokines IL-10 and TGF-β, indicating a M2-like phenotype [46]. In addition, a higher expression of IL-10 and TGF-β cytokines was demonstrated in OSCC as compared to biopsies from normal oral mucosa [46]. TAMs were found in increased quantities in metastatic vs. non-metastatic OSCC specimens [46]. Moreover, numerous retrospective studies have shown a correlation of TAMs with poor outcomes in OSCC [47,48,49,50,51,52]. In a more recent study, TAMs were detected in higher percentages in tumor biopsies from patients with OSCC and no history of smoking or alcohol consumption [53]. Interestingly, abundance of TAMs was correlated with absence of cancer stem cell (CSC) markers (NANOG and SOX2) and high PD-L1 expression, indicating a possible involvement in immune escape [53]. However, in another study, TAMs have been significantly correlated with CSC markers, such as SOX2 and ALDH1 [51]. A possible link between TAMs and propulsion of epithelial to mesenchymal transition (EMT) has been also demonstrated in OSCC [54]. Finally, in a study that evaluated surgical biopsies of patients with low grade dysplasia, high grade dysplasia and OSCC, the percentage of TAMs was found to rise through the steps of cancer progression; of note, they were found in higher density in HPV-positive (HPV+) compared to HPV-negative (HPV-) individuals [52].

Given their multifaceted role in creating an immunosuppressive TME, targeting TAMs has emerged as an intriguing strategy in HNSCC. Treatment strategies are focused either on inhibition of TAM recruitment or reversal of polarization of TAMs. Chemoattractant chemokine ligand (CCL2), which has been studied more extensively, has been shown to correlate with high infiltration of TAMs and metastatic lymph node involvement in OSCC [55]. In addition, the production of CCL2 by HNSCC educates monocytes into M2 macrophages [56]. In an experimental model, the inhibition of CCL2 production by curcumin has been shown to block the evasion of HNSCC cells [56]. Although CCL2 inhibitors, such as carlumab and PF04136309 have yielded promising results in pancreatic and other cancers [57,58], their use has not been investigated in HNSCC. Blockade of CSF1R, a growth factor involved in TAM migration and recruitment, has been also explored as a therapeutic strategy. Ries et al. constructed RG7155, a monoclonal antibody against CSF1R, which was shown to decrease levels of TAMs with a concomitant elevation of T cells in animal models [59]. Subsequent use of the drug in patients demonstrated a reduction in CSF1R(+) CD163(+) macrophages in biopsies and objective response in patients with giant cell tumors [59]. In a phase I trial, RG7155, which is marketed under the name emactuzumab, administered alone or in combination with paclitaxel, resulted in a significant reduction of immunosuppressive TAMs, which did not, however, translate into clinically relevant responses in patients [60]. Nevertheless, emactuzumab is being investigated in combination with immune checkpoint inhibitor atezolizumab in a phase I trial in advanced solid tumors (NCT0232319). Pexidartinib, a novel CSF1R inhibitor that is currently FDA approved for tenosynovial giant cell tumor is also being evaluated in advanced solid tumors in a phase I trial (NCT02734433).

A second therapeutic strategy that aims to eliminate TAMs is reversing polarization from a phenotype with immunosuppressive activity (M2) to a phenotype with tumoricidal properties (M1). Several approaches to achieve repolarization of TAMs are toll-like receptor (TLR) agonists, CD40 agonists and inhibition of PI3Kγ [61]. TLRs are cell surface receptors that identify pathogen-associated components and have to capacity to provoke polarization of TAMs towards a M1 cytolytic phenotype [61]. Indeed, in experimental HNSCC mouse models, intrarumoral injection of TLR7 and TLR9 agonists in combination with PD-1 blockade led to an increase of M1/M2 macrophage ratio, prompted the recruitment of CTLs and resulted in tumor regression [62]. TLR 7/8 agonist imiquimod, which is administered topically, is currently being tested in combination with 5-FU in squamous cell carcinoma of the lower extremities (NCT03370406). Similarly, TLR9 agonist SD-101, which is given as an intratumoral injection, has been evaluated in a phase I/II trial in combination with pembrolizumab in advanced, immunotherapy-naïve HNSCC. Among 23 patients, five responded and six had stable disease, revealing a 48% DCR in this cohort. The combination was well tolerated [63]. An ongoing phase I study is currently evaluating immune response (measured by the number of CD8+ T cells pre-treatment and post-surgery) produced by the combination of nivolumab and TLR8 agonist motolimod in patients with resectable HNSCC (NCT03906526).

CD40 belongs to the tumor necrosis factor (TNF) family of receptors and inverts M2 macrophages into M1, resulting in production inflammatory cytokines [64]. A phase I/II study of the CD40 agonist selicrelumab in combination with atezolizumab in metastatic solid tumors has been completed and results are awaited (NCT02304393). In another phase I study, the CD40 agonist CDX-1140 is being evaluated as monotherapy or in combination with pembrolizumab, chemotherapy or the recombinant fms-like tyrosine kinase 3 (Flt-3) ligand CDX-301 in advanced solid tumors including HNSCC (NCT03329950). Finally, PI3Kγ is a kinase that induces T cell inactivation; successful inhibition leads to secretion of inflammatory cytokines in TAMs and tumor regression in preclinical models [65]. The combined PI3Kγ and PI3kδ inhibitor duvelisib has been recently approved for hematological malignancies [66]. IPI-549, a selective PI3Kγ inhibitor, has shown clinical activity in combination with nivolumab in a phase I trial [67]. Of note, IPI-549 is currently being assessed as monotherapy in resectable HNSCC (NCT03795610).

### 3.2. Cancer-Associated Fibroblasts (CAFs)

CAFs represent a dominant population of cells in the TME, which is phenotypically and functionally distinct from normal fibroblasts [68]. Upon tissue trauma, normal fibroblasts are activated, become smooth muscle reactive fibroblasts (myofibroblasts) and have a key role in wound healing and control of inflammation. CAFs are myofibroblasts with proliferative and migratory characteristics, which enable them to produce a diversity of molecules that enhance tumor proliferation, remodel the extracellular matrix (EMT) that leads to tissue stiffening, incite tumor evolution and metastasis and influence the immune response [69,70]. Although their origin has not been entirely clarified, it is suggested that they are either descendants of bone marrow-derived mesenchymal stem cells (MSCs), or products of education of fibroblasts by tumor cells [71,72]. Other possible theories include cytokine-mediated differentiation of fibroblasts and malignant transformation of fibroblasts by hypoxia-inducible factor (HIF) 1a transcription factor [73,74]. CAFs are usually identified by the expression of α-smooth muscle actin (α-SMA), which is also positive in activated fibroblasts at the site of a tissue injury [75]. However, a variety of other biological markers, such as FAP, S100A4 and platelet derived growth factor receptor-β (PDGFRβ) are co-expressed and although they are non-specific for this cell population, they used in combination to identify CAFs [76].

CAFs have been implicated in HNSCC progression and metastasis. In a recent study, it was shown that CAFs produce the matrix-specific protein periostin, which is upregulated in HNSCC tissues [77]. In addition, exogenous periostin reinforces the proliferation and invasion of HNSCC cells [77]. In another study, invasiveness of HNSCC cell lines was associated with expression of periostin [78]. Interestingly, several studies have demonstrated a substantial correlation between α-SMA-positive CAFs with survival in patients with OSCC [79,80,81,82,83]. A recent meta-analysis which included immunohistochemical studies assessing the prognostic significance and clinical relevance of CAFs revealed that the presence of elevated levels of CAFs in tumor stroma correlated with short disease-free survival (DFS) and OS [84]. In addition, a high quantity of CAFs was associated with parameters of tumor aggressiveness, such as advanced stage, grade and vascular invasion [84]. A more recent meta-analysis of 11 immunohistochemical studies confirmed these results, showing an increased mortality risk in patients with high CAF density in their tumor samples [85].

Due to their dominance in the TME, mobility and flexibility, CAFs have the great advantage of being easily isolated and cultured [86]. However, they tend to be a rather phenotypically and functionally heterogeneous cell population. Notably, the recognition of specific CAF markers has enabled to distinguish subpopulations with tumor-promoting properties and could facilitate the discovery of CAF-targeted therapies. CAF-directed anticancer treatments generally focus on exhaustion of CAFs via genetic deletion or pharmaceutical inhibition of cell surface markers, alteration of CAF activation or function through targeting of chemokines, normalization and inactivation of CAFs, targeting of CAF-derived ECM and use of CAFs as a vehicle for drug delivery, such as oncolytic adenoviruses [86]. Research within this field in HNSCC is somewhat poorly developed. Nevertheless, many of these molecules have been shown to be involved in HNSCC carcinogenesis and prognosis, and the investigation of new targeted therapies is awaited [87,88,89].

Although α-SMA represents the most identifiable marker of CAFs, inhibition of myofibroblasts via α-SMA targeting has yielded surprising results. Ozdemir et al. used a genetically engineered mouse model of pancreatic carcinoma in which α-SMA positive CAFs were pharmaceutically depleted. Depletion of α-SMA expressing CAFs resulted in aggressive, hypoxic tumors and correlated with poor survival [90]. On the other hand, Fibroblast Activation Protein (FAP) is overexpressed in activated stromal fibroblasts, has a key role in ECM remodeling and regulates proliferation and migration of CAFs [91]. In xenograft models of lung, pancreas and HNSCC, treatment with a novel anti-FAP monoclonal antibody-maytansinoid conjugate known as FAP5-DM1, conferred durable suppression of tumor development with good tolerability [92]. However, in a phase I escalation trial that included 26 patients with lung and colorectal cancer, the administration of the anti-FAP monoclonal antibody sibrotuzumab did not result in any objective response [93]. RO6874281, an immunocytokine comprising of an interleukin-2 variant fused with an anti-FAP antibody, has been shown to increase T and NK cells and produce a durable response in one patient with HNSCC in a phase I trial [94]. An ongoing phase II basket study which is evaluating RO6874281 in combination with atezolizumab in solid tumors, including ICI-naïve or previously treated HNSCC, is currently recruiting patients (NCT03386721).

Targeting cytokines and other factors that are implicated in CAF biology is also a tempting approach. Among them, IL-6 and JAK2/STAT3 pathways represent promising targets involved in the activation of CAFs. IL-6 is secreted by several immune cells [95] and has been shown to correlate with aggressive behavior and poor outcomes in HNSCC [96,97]. Most importantly, IL-6 leads to the activation of STAT3 and overexpression of downstream tumor-suppressor genes in HNSCC, which subsequently propels the reinforcement of tumor cell expansion and migration [98]. Despite positive results in preclinical models [99], Siltuximab, a monoclonal antibody against IL-6 has failed to produce objective responses in a phase I/II clinical trial that included patients with advanced solid tumors including HNSCC [100]. On the other hand, development of STAT3 inhibitors has been difficult through the years due to constitutive activation of STAT3 by a variety of signaling pathways and other factors [101]. Notably, C188-9 a small STAT3 inhibitor, has been shown to achieve tumor regression in mice with xenografts of radioresistant HNSCC lines [102]. C188-9 is being investigated in a phase I trial in advanced solid tumors including HNSCC, which is currently recruiting patients (NCT03195699). Finally, JAK2 inhibitors have shown to decrease tumor progression in patient-derived xenografts from HNSCC tumors [103]. Ruxolitinib, a JAK1/2 inhibitor approved for myelofibrosis, is currently being evaluated in operable HNSCC (NCT03153982).

ECM remodeling mediated by CAFs is one of the responsible mechanisms of increased desmoplastic stroma that enhances tumor progression and hampers the delivery of therapeutic drugs in the tumor bed [86]. Matrix Metalloproteinases (MMPs) are calcium-dependent endoproteases that degrade collagen and are implicated in ECM remodeling. S-3304, a potent, oral MMP inhibitor has been evaluated in a phase I pharmacokinetic study that enrolled patients with advanced solid tumors, including two patients with HNSCC [104]. Although the drug was shown to be tolerable, no objective responses were identified. However, among seven patients with stable disease, one had HNSCC [104].

### 3.3. Tregs

Tregs represent a subgroup of CD4+ T cells that are characterized by the expression of CD25, transcription factor forkhead box P3+ (FOXP3+), CTLA4, glucocorticoid-induced TNF receptor (GITR) and OX40 (CD134) [34]. They typically repress immune response to avoid exaggerated immune reactivity by imposing suppressive activity on CTLs and CD4 helper cells, and are involved in immune escape by secreting immunosuppressive cytokines, such as IL-10 and TGF-β, contributing critically to an immunosuppressive milieu [105]. Normally, Tregs originate in the thymus and are afterwards recruited in the TME [38]. Using tumor-bearing mice, Valzasina et al. showed that cancers have the ability to expand Tregs in the periphery, by converting CD4+CD25− T cells into Tregs [106]. In addition, IL-10 and TGF-β mediate the conversion of FOXP3(−) T cells into the TME [107]. Several chemokines, such as CCR4, play a major role in recruitment of Tregs to the TME [108].

Emerging clinical evidence has demonstrated an elevated level of Tregs both in the blood and tumor tissue of patients with HNSCC [109,110,111,112,113,114]. Saloura et al. showed that only HPV (+) tumor tissues are enriched in Treg markers [115]. Several studies have documented that circulating Treg levels correlate with advanced stage of disease and aggressive pathological features [110,116]. Interestingly, Schaefer et al. demonstrated that both patients with active disease and those with complete response after treatment have a high frequency of circulating Tregs, indicating that disruption of immune homeostasis in the periphery is not restored following successful treatment [113]. In contrast, Strauss et al. found a higher frequency of circulating Tregs in patients with no evidence of disease as compared to patients with active disease [117].

There is no clear consensus in the literature regarding the prognostic significance of T regs in HNSCC. A handful of studies have suggested a correlation of increased Treg levels either in circulation or tumor tissue with poor prognostic outcomes [109,116,118,119]. However, several studies have shown an association of tumor infiltrating Tregs with favorable survival and better locoregional control [114,120,121]. This finding might be considered a paradox, since evidence suggests that infiltrating Tregs are more immunosuppressive in HNSCC as compared to circulating Tregs [122,123]. These controversies might partly reflect the inconsistency of a universal definition of Tregs, since the majority of studies report exclusively on FOXP3(+) cells [34]. In addition, biological heterogeneity might play a role, since Tregs display distinct characteristics determined by primary tumor location, histology, and molecular profile [105].

Therapeutic approaches of successful Treg targeting mainly rely on blockade of surface markers, such as CD25, FOXP3, chemokine receptors, OX40, and GITR, as well as the inhibition of immunosuppressive cytokines that could repress Treg function. CD25 represents an intriguing target for depletion of Tregs. Oweida et al. used a preclinical murine model of HNSCC and showed that concurrent administration of RT, inhibitors against immune checkpoints PD-L1 and T-cell immunoglobulin mucin-3 (TIM-3) and a monoclonal anti-CD25 antibody led to a more durable therapeutic response and tumor regression as compared to treatment with RT in combination only with anti-PD-L1/anti-TIM-3. Indeed, the administration of a Treg inhibitor resulted in exhaustion of the revived Tregs that were shown to be the cause of treatment resistance [124]. A major issue with CD25 targeting is that it is not selectively expressed on Tregs and treatment with anti-CD25 antibodies might result in co-depletion of activated effector cells interfering with the therapeutic goal, as depicted in a phase I/I study of patients with melanoma, where the administration of the anti-CD25 antibody daclizumab in combination with a dendritic vaccine failed to produce any clinical responses [125]. Targeting FOXP3 expression might be feasible via the reversal of Treg-specific demethylated region (TSDR) demethylation, a natural process that mediates FOXP3 stability, but research in this field remains experimental [126]. AZD8701, a recently developed antisense oligonucleotide inhibitor of FOXP3 has shown promising activity in preclinical mouse models both as monotherapy or in combination with immune checkpoint blockade [127]. AZD8701 is currently being evaluated as monotherapy or in combination with durvalumab in a phase I study recruiting patients with advanced solid tumors including HNSCC (NCT04504669).

Targeting chemokine receptors, such as CCR4, which is implicated in Treg chemotaxis and function constitutes a promising treatment approach. In melanoma, the ex vivo depletion of CCR4(+) T cells from the peripheral blood of patients and subsequent in vitro stimulation of the depleted cell population with a tumor antigen, led to augmentation of antigen-specific CD4(+) T cells [128]. Mogamulizumab, a humanized antibody against CCR4, has been successful in achieving Treg exhaustion and a 40% disease control in a phase Ia study that included 10 patients with lung and esophageal cancer [129]. When used in combination with anti-PD-L1 antibody durvalumab or anti-CTLA-4 antibody tremelimumab, mogamulizumab failed to show significant efficacy in a phase I trial that included patients with advanced solid tumors, including HNSCC [130]. On the other hand, a phase I study that included 96 patients with advanced solid tumors revealed a favorable toxicity profile and good antitumor activity (27% ORR) of the mogamulizumab/nivolumab combination [131]. Mogamulizumab is currently being evaluated in clinical trials in combination with ICIs in advanced solid tumors in various settings (NCT02705105, NCT02946671, NCT02301130).

OX40 is a co-stimulatory receptor that is known to promote the proliferation and memory of cytotoxic T cells [132]. In HNSCC, OX40 has been shown to be highly upregulated in tumor infiltrating T regs [133]. In preclinical models of murine tumors, stimulation of the OX40 receptor using either a ligand or an agonist resulted in prolongment of survival [134]. In addition, OX40 engagement has been shown to improve survival and reduce tumor recurrence when combined with radiation or surgical resection in mouse models [135]. A murine agonistic anti-OX40 antibody has been evaluated in a phase I study in patients with advanced cancer, showing clinical regression of metastatic lesions, increased T and B responses and increased OX40 expression in Tregs [136]. MEDI0562, an agonistic humanized anti-OX40 antibody, demonstrated promising efficacy in a phase I trial conducted in 55 patients with advanced solid tumors, including 26 patients with HNSCC. More specifically, the drug provoked increased effector T cell proliferation and reduction of itratumoral OX40 (+) Tregs, while two patients had PR and 44% had stable disease [137]. MEDI0562 is currently being assessed in HNSCC in the neoadjuvant setting (NCT03336606). MEDI6469, a murine anti-OX40 monoclonal antibody is also being evaluated in resectable HNSCC (NCT02274155). In a preliminary report that included 10 patients, MEDI6469 was successful in inducing effector T cell activation and upregulation of PD-L1 [138]. A phase I trial of MEDI6469 alone or in combination with tremelimumab or durvalumab in metastatic solid tumors was terminated early at the sponsor’s discretion (NCT02205333). Another human agonistic OX40 antibody, MEDI16383, is currently being tested in a phase I clinical trial alone or in combination with durvalumab in patients with recurrent or metastatic solid tumors (NCT02221960). In addition, INBRX-106, a recombinant, humanized, hexavalent antibody that stimulates OX40, is being tested in a phase I study in combination with pembrolizumab in patients with pretreated locally advanced or metastatic solid tumors, including HNSCC (NCT04198766).

Other strategies aimed at Tregs include stimulation of co-stimulatory receptor GITR, which is commonly expressed on Tregs and inhibition of CD39, CD73 and adenosine pathway. In tumor-bearing mice, administration of an agonistic GITR antibody results in tumor regression and increased infiltration by effector T cells [139]. Increased efficacy was observed with concurrent administration of an anti-CTLA-4, but not anti-CD25 antibody [139]. MEDI1873, a novel GITR-ligand/IgG1 agonist was recently evaluated in a phase I study in patients with advanced solid tumors, including HNSCC. Interestingly, the drug induced an intratumoral decrease of FOXP3+GITR+ T cells and resulted in stable disease in 42.5% of patients with an acceptable toxicity profile [140]. REGN6569, a novel anti-GITR antibody, is currently being tested in advanced HNSCC in combination with the PD-1 inhibitor Cemiplimab (NCT04465487). Finally, targeting CD73, CD39, and adenosine-induced immunosuppressive activity has yielded promising results in preclinical models [141,142]. Indeed, oleclumab, a monoclonal antibody that targets CD73, acts by inhibiting adenosine-mediated lymphocyte repression, resulting in activation of effector T cells and macrophages and decrease of Tregs and MDSCs. In a phase I trial in colorectal and pancreatic carcinoma, the combination if oleclumab and durvalumab was tolerable and yielded propitious efficacy [143]. This combination is being evaluated in a phase II trial in recurrent metastatic HNSCC, NSCLC, and pancreatic cancer (NCT04262388).

### 3.4. MDSCs

MDSCs are defined as a highly heterogeneous group of immature myeloid cells that are normally found on low quantities and participate in immune response and tissue regeneration [144]. Two principal categories are present in humans, polymorphonuclear MDSCs (PMN-MDSCs) and monocytic MDSCs (M-MDSCs), which are phenotypically and functionally different [34]. During carcinogenesis, MDSCs rapidly increase and are recruited in the TME, where they display an immunosuppressive behavior though promotion of neovascularization, repression of CTLs, disruption of antigen presentation machinery, differentiation into TAMs and alteration of NK function [145]. Mobilization and migration of MDSCs to the TME is accomplished by chemokine ligand CXCL1, recombinant human granulocyte macrophage colony-stimulating factor (GM-CSF), IL-8, and CSF1 [38]. In addition, PMN-MDSCs express chemokine receptors CXCR2 and CSFR1 [96].

Human MDSCs are characterized as CD14−CD11b + CD33+ without expression of the MHC Class-II molecule HLA-DR, which is traditionally expressed in mature myeloid and lymphoid cells [146]. Tumor infiltrating MDSCs are difficult to assess in tissue samples due to lack of a uniform identification, the presence of complex surface markers and the use of various detection methods [34,147]. In HNSCC, intratumoral MDCSs have been correlated with tumor recurrence [119], advanced stage, adverse outcomes and malignant progression [148]. Regarding MDSCs in peripheral blood, a meta-analysis of 7 studies that included patients with mainly gastrointestinal cancers, breast cancer and melanoma demonstrated a negative prognostic role of circulating MDSCs [149]. Similar findings were observed in a larger met-analysis of 40 studies in patients with advanced solid tumors [147]. In HNSCC, high levels of circulating MDSCs were associated with advanced stage and poor outcomes in one study [150].

Given the pluralistic role of MDSCs in oncogenesis, a variety of therapeutic approaches targeting MDSCs are investigated, which focus either on prevention of MDSC differentiation, inhibition of MDSC activation or blockade of MDSC immunosuppression. First, MDSCs have the capacity to promote immunosuppression through IDO1 expression [151]. In HNSCC, high IDO1 expression has been correlated with worse outcomes in retrospective studies [152,153]. In the phase I/II ECHO-202/Keynote 037 study, which combined the IDO1 inhibitor epacadostat with pembrolizumab, among 2 patients with HNSCC who were included, one patient had stable disease as best response [154]. However, a phase III trial in patients with melanoma failed to show any survival advantage of the combination versus epacadostat monotherapy [155]. A phase III study comparing epacadostat/pembrolizumab combination or pembrolizumab monotherapy to the standard EXTREME regimen (Keynote 669/Echo 304) in R/M HNSCC has been completed and results are awaited (NCT03358472). Notably, the KEO study, designed to evaluate the combination of epacadostat and pembrolizumab as a neoadjuvant treatment in resectable HNSCC was withdrawn and never opened to enrollment NCT03325465). On the other hand, the combination of IDO1 inhibitor navoximod with atezolizumab has demonstrated good tolerability and antitumor activity in patients with solid tumors including one patient with HNSCC in a phase I study [156]. Finally, BMS-986205, an oral IDO1 inhibitor that has demonstrated encouraging activity in combination with nivolumab in advanced urothelial cancer [157], is being assessed in an ongoing phase II window of opportunity trial in patients with stage II-IV HNSCC in combination with nivolumab (NCT03854032). The study is currently recruiting patients.

Second, blockade of the chemokine receptor CXCR2, which is expressed on MDSCs represents a tempting approach. In head and neck and lung tumor-bearing mice, treatment with SX-682, a small inhibitor of both CXCR1 and CXCR2, resulted in abrogation of tumor infiltrating MDSCs. In addition, combination treatment with anti-PD-1 blockade reinforced tumor regression [158]. In other study, administration of SX-682 enhanced activity of NK cells in HNSCC preclinical models [159]. Finally, inhibition of phosphodiesterase 5 (PDE5), which functionally inactivates MDSCs, is a promising novel approach. In a phase II trial conducted in patients with HNSCC, the PDE5 inhibitor tadalafil was shown to enhance antitumor immunity by promoting T cell expansion and decreasing MDSCs in the periphery [160]. A second phase II study is evaluating assessing the efficacy of tadalafil in patients with resectable or locally advanced disease treated with definitive therapy (NCT01697800). Tadalafil is also being tested in combination with a vaccine in resectable recurrent disease (NCT02544880).

**Figure 1:**
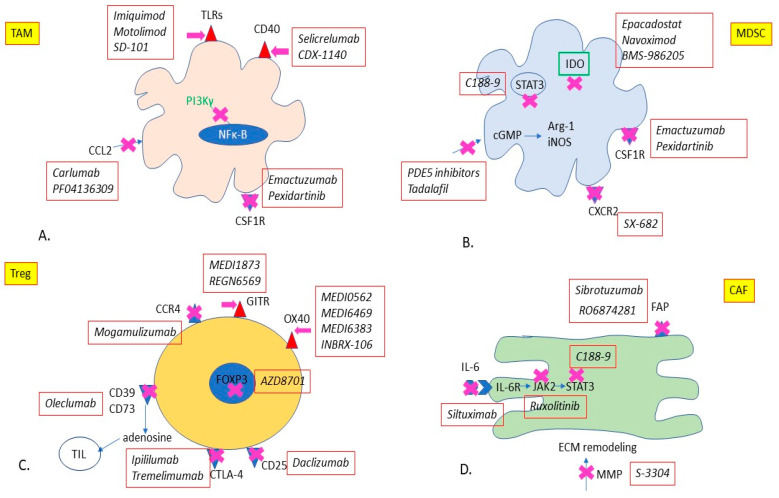
Therapeutic strategies for TME-directed therapy. (**A**) Targeting TAMs: (1) Anti-CCL2: Monoclonal antibody Carlumab, CCL2 antagonist PF04136309, (2) Anti-CSF1R: Monoclonal Antibody Emactuzumab, tyrosine kinase inhibitor Pexidartinib, (3) TLR agonists: small molecules imiquimod, motolimod, SD-101, (4) CD40 agonists: Monoclonal Antibodies Selicrelumab and CDX-1140, PI3Kγ inhibitor: IPI-549. (**B**) Targeting MDSCs: (1) IDO inhibitors: Epacadostat, Navoximod, BMS-986205, (2) Anti-CSF1R: Monoclonal Antibody Emactuzumab, tyrosine kinase inhibitor Pexidartinib, (3) CXCR2 inhibitor: SX-682 (4) PDE5 inhibitor tadalafil, (5) STAT3 inhibitor C188-9. (**C**) Targeting Tregs: (1) GITR agonists: Monoclonal Antibodies MEDI1873 and REGN6569, (2) OX40 agonists: Monoclonal Antibodies MEDI0562, MEDI6469, MEDI6383 and INBRX-106, (3) FOXP3 inhibitor: AZD8701, (4) Anti-CD25: Monoclonal Antibody Daclizumab, (5) Anti-CTLA-4: Monoclonal Antibodies Ipilimumab and Tremelimumab, (6) Anti-CD73: Monoclonal Antbody Oleclumab, (7) Anti-CCR4: Monoclonal Antibody Mogamulizumab. (**D**) Targeting CAFs: (1) Anti-FAP: Monoclonal Antibodies Sibrotuzumab and RO6874281, (2) STAT3 inhibitor: C188-9, (3) JAK2 inhibitor: Ruxolitinib, (4) Anti-IL-6: Monoclonal Antibody Siltuximab, (5) Anti-MMP: small molecule S-3304. Abbreviations: CAFs = Cancer Associated Fibroblasts, CCR4 = C-C Motif Chemokine Receptor 4,CD73 = Cluster of Differentiation 73, CD40 = Cluster of differentiation 40, CSF1R = Colony stimulating factor, receptor, CTLA-4 = Cytotoxic T Lymphocyte-Associated protein 4,FAP = Fibroblast Activation Protein, FOXP3 = Forkhead box P3, GITR = Glucocorticoid-Induced TNFR-related protein, IDO1 = Indoleamine 2,3-dioxygenase 1, JAK2 = Janus Kinase 2, MDSCs = Myeloid-Derived Suppressor cells, PDE5 = Phosphodiesterase 5, PI3Kγ = Phosphoinositide 3-Kinase γ, STAT3 = Signal transducer and activator of transcription 3, TAMs = Tumor Associated Macrophages, TLR = Toll-like Receptor, Tregs = T regulatory cells.

Figure 1 illustrates therapeutic strategies for targeting TAMs, CAFs, Tregs, and MDSCs.

### 3.5. Angiogenesis

The theory of angiogenesis, which underpins the importance of new vessel formation in tumors due to the increasing need for oxygen and nutrients, was initially postulated by Folkman in 1971 [161]. Since then, meticulous research on this field has established angiogenesis as a well-known hallmark of cancer [162] and has integrated antiangiogenic agents in the standard treatment combinations of various advanced solid tumors.

Enhanced tumor vascularization involves the coordination of several cellular components of the TME, such as vascular endothelial cells and pericytes; in addition, immunosuppressive cell types, including TAMs and CAFs promote neovascularization by secreting pro-angiogenic factors [145,163]. Vascular endothelial factor (VEGF), a key protein that propels endothelial cell proliferation, is upregulated by inflammatory chemokines, such as CXCL12 [23]. VEGF overexpression has been associated with aggressive disease and poor outcomes in HNSCC [164,165].

Inflammation and hypoxia give impetus to angiogenenesis. Chronic inflammation in the TME, which is reinforced by the transcription factor NF-κβ, contributes to the production of angiogenic factors [23]. Intratumoral hypoxia is generated by tumors that display expansionary properties and decrease blood flow [69]. Hypoxia in HNSCC is associated with radiotherapy and chemotherapy resistance and contributes to immune escape by interfering with the function of TAMs and T lymphocytes [166].

Antiangiogenic therapy has been shown to increase permeability of drugs into the tumor by decreasing interstitial fluid pressure [167]. The monoclonal antibody against VEGF bevacizumab in combination with chemotherapy has become the standard of care in several advanced solid tumors. In HNSCC, preclinical studies in murine models have demonstrated that treatment with sunitinib, a multi-tyrosine kinase inhibitor that targets VEGFR, leads to a reduction of tumor microvessel density with subsequent tumor regression and oxygenation [168]. Despite promising preclinical activity, sunitinib failed to show any efficacy as monotherapy in advanced HNSCC [169,170]. Furthermore, the combination of bevacizumab with chemotherapy did not improve OS as compared to chemotherapy alone in a phase III study that included patients with recurrent/metastatic (R/M) HNSCC [171].

The rationale for combining anti-angiogenics with immunotherapy is based on the immunosuppressive properties of VEGF, which has been shown to decrease T cell recruitment and infiltration into the tumor [18], reduce adhesion of immune effector cells to tumor vessels [172], hampers T cell function [173], affects the functional capacity of dendritic cells [174] and enables the accumulation of Tregs [175]. In a phase I trial that included patients with advanced gastric cancer, NSCLC, and urothelial carcinoma, the combination of pembrolizumab with the monoclonal antibody against VEGFR ramucirumab yielded satisfactory responses and had a good safety profile [176]. On the other hand, lenvatinib is a multiple kinase inhibitor that acts against VEGFR1, VEGFR2 and VEGFR3 and is currently FDA approved as monotherapy for hepatocellular cancer and thyroid carcinoma. A phase Ib/II trial that evaluated the addition of lenvatinib to pembrolizumab in patients with advanced solid tumors including HNSCC, revealed a 36% RR in HNSCC with manageable adverse events [177]. LEAP-10 (NCT04199104) is a randomized phase III study comparing pembrolizumab plus lenvatinib to pembrolizumab plus placebo as first-line treatment in patients with R/M HNSCC whose tumor is associated with CPS PD-L1 score ≥ 1. The primary endpoints of LEAP-10 include ORR, PFS and OS. This combination of lenvatinib plus pembrolizumab has already been FDA approved for previously treated, advanced endometrial cancer. Several ongoing studies assess combinations of angiogenesis inhibitors with immunotherapy in R/M HNSCC, such as ramucirumab/pembrolizumab and bevacizumab/atezolizumab (NCT03650764, NCT03818061).

Table 1 summarizes ongoing clinical trials evaluating novel pharmaceutical agents targeting the TME, either alone or in combination with immunotherapy.

## 4. Conclusions

The TME is a dynamic territory, which is reformed by the tumor to its own advantage. Analysis of the TME has emerged as an exciting field of research with the goal of discovering novel predictive biomarkers of response to immunotherapy. New biological concepts have been revealed through the meticulous study of TME components that have provided a foundation for novel therapeutic approaches in order to create a tumor-inflamed phenotype. Immunosuppression within the TME is largely manipulated by heterogeneous populations of cells, such as TAMs, CAFs, MDSCs and Tregs and successful targeting of these cells by inhibiting their activation or function might reverse local immunosuppression, providing a breeding ground for augmentation of antitumor immunity and reversing treatment resistance. Several clinical trials have yielded promising results. Further clinical trials will shed light on the optimal way to overcome the barriers of the TME.

## Figures and Tables

**Table 1:** Ongoing clinical trials targeting the TME ± immunotherapy in HNSCC.

Trial NCT/Name	N of Pts	Phase	Stage/Eligibility	Treatment	Target	Primary Endpoint
TAMs						
NCT02323191	221	I	Advanced solid tumors	Emactuzumab + Atezolizumab	CSF1R PD-L1	% of pts with DLTs MTD of emactuzumab % of pts with AEs
NCT02526017	295	I	Advanced Solid tumors (including a HNSCC cohort)	Cabiralizumab + Nivolumab	CSF1R PD-1	Safety RD of cabiralizumab ORR
NCT03906526	72	I	Untreated, resectable HNSCC	Motolimod + Nivolumab, motlimod monotherapy, nivolumab monotherapy	TLR8 PD-1	Number of CD8+ T cells pre-treatment and post-surgery
NCT02304393	140	Ib	Advanced solid tumors	Selicrelumab + Atezolizumab	CD40 PD-L1	Safety, MTD, DLTs, PFS, ORR, OS
NCT03329950	260	I	Advanced Solid tumors (including a HNSCC cohort)	CDX-1140, CDX-1140+ pembrolizumab or chemotherapy or CDX-301	CD40 PD-1	Safety and tolerability
NCT03795610		IΙ	Resectable HNSCC	IPI-549	PI3Kγ	PI3Kγ changes
CAFs						
NCT03386721	322	II	Advanced HNSCC, NSCLC, squamous esophageal and cervical cancers	RO6874281 + atezolizumab	FAP PD-L1	ORR
NCT03195699	30	I	Advanced Solid tumors (including a HNSCC cohort)	C118-9	STAT3	MTD and pharmacokinetics
NCT03153982	45	II	Resectable HNSCC	Ruxolitinib	JAK2	Changes in tumor size
Tregs						
NCT04504669	123	I	Advanced Solid tumors (including a HNSCC cohort)	AZD8701 ± durvalumab	FOXP3 PD-L1	MTD, AEs, ORR
NCT02946671	16	I	Resectable cancers, including oral cancer	Mogamulizumab+ Nivolumab	CCR4 PD-1	AEs, FOXP3 (+) tumors
NCT02301130	64	I	Advanced solid tumors	Mogamulizumab + Durvalumab or Mogamulizumab + Tremelimumab	CCR4 PD-L1 CTLA-4	Safety
NCT02705105	114	I/II	Advanced solid tumors	Mogamulizumab + Nivolumab	CCR4 PD-1	MTD, DLT
NCT02274155	17	Ib	Resectable stage III-IV HNSCC	MEDI6469	OX-40	Safety and feasibility of surgical resection
NCT03336606	35	Ib	Advanced resectable HNSCC, Stage IIIB/IIIC melaonoma	MEDI0562	OX-40	Immune activation
NCT02221960	39	I	Recurrent/Metastatic solid tumors	MEDI6383	OX-40 PD-L1	Safety
NCT04198766	150	I	Advanced Solid tumors (including a HNSCC cohort)	INBRX 106 +/- permbrolizumab	OX-40 PD-1	Safety, MTD
NCT04465487	75	I	Advanced Solid tumors (including a HNSCC cohort)	REGN6569 + Cemiplimab	GITR PD-1	Safety, DLTs, ORR, percentage change in GITR/Treg density
NCT04262388	120	II	Recurrent/Metastatic HNSCC, NSCLC and pancreatic cancer	Oleclumab + durvalumab	CD73 PD-L1	mRNA-seq based assay of blood samples, toxicity, ORR, DCR, DoR
MDSCs						
NCT03358472	89	III	R/M HNSCC	Epacadostat + Pembrolizumab vs Pembrolizumab vs EXTREME regimen	IDO1 PD-1	ORR
NCT03854032	48	II	Stage II-IV HNSCC	BMS-986205 + nivolumab	IDO1 PD-1	ORR
NCT01697800	40	II	Resectable or locally advanced HNSCC	Tadalafil vs placebo	PDE5	Change in immune response
NCT02544880	16	I/II	Resectable recurrent or second primary HNSCC	Tadalafil + anti-MUC1 vaccine + anti-influenza vaccine	PDE5	Safety Tumor-specific immune response
Angiogenesis						
NCT03818061	110	II	R/M HNSCC	Bevacizumab +Atezolizumab	VEGF PD-L1	ORR
NCT03650764	42	I/II	R/M HNSCC	Ramucirumab + Pembrolizumab	VEGFR2 PD-1	RD of Ramurcirumab ORR
NCT04199104	500	III	PD-L1 (+) R/M HNSCC	Lenvatinib + Pembrolizumab vs Pembrolizumab	VEGFR1,2,3 PD-1	ORR, PFS, OS
T cells						
NCT04128696	600	II/III	PD-L1 (+) R/M HNSCC	GSK3359609 + Pembrolizumab vs. Pembrolizumab	ICOS PD-1	OS and PFS in PD-L1 CPS ≥ 1, OS in PD-L1 CPS ≥ 20
NCT04428333	640	II/III	R/ HNSCC	GSK3359609 + Pembrolizumab+ 5-FU/platinum vs. Pembrolizumab + 5-FU/platinum	ICOS PD-L1	OS and PFS in total population, PFS in PD-L1 CPS ≥ 1

AE = Adverse Event, CAFs = Cancer Associated Fibroblasts, CCR4 = C-C Motif Chemokine Receptor 4,CD73 = Cluster of Differentiation 73, CD40 = Cluster of differentiation 40, CSF1R = Colony stimulating factor, receptor, CTLA-4 = Cytotoxic T Lymphocyte-Associated protein 4, DCR = Disease Control Rate, DLT = Dose Limiting Toxicity, DoR = Duration of Response, FAP = Fibroblast Activation Protein, FOXP3 = Forkhead box P3, GITR = Glucocorticoid-Induced TNFR-related protein, HNSCC = Head and Neck Squamous Cell Carcinoma, ICOS = T-cell co-stimulator, IDO1 = Indoleamine 2,3-dioxygenase 1, JAK2 = Janus Kinase 2, MDSCs = Myeloid-Derived Suppressor cells, MTD = Maximum Tolerated Dose, MUC-1 = Mucin-1, cell surface associated, N = Number, NSCLC = Non-Small Cell Lung Cancer, ORR = Overall Response Rate, OS = Overall Survival, PD-1 = Programmed Death-1, PD-L1 = Programmed Death-1 Ligand, PDE5 = Phosphodiesterase 5, PFS = Progression Free Survival, PI3Kγ = Phosphoinositide 3-Kinase γ, Pts = Patients, RD = Recommended Dose, R/M = Recurrent/Metastatic, STAT3 = Signal transducer and activator of transcription 3, TAMs = Tumor Associated Macrophages, TLR8 = Toll-like Receptor 8, Tregs = T regulatory cells, VEGF = Vascular Endothelial Growth Factor, VEGFR = Vascular Endothelial Growth Factor Receptor.
